# Comparative efficacy and safety of ciprofol, propofol, and propofol and etomidate mixture, and ciprofol and etomidate mixture in patients undergoing painless gastroscopy: A randomized, double-blind controlled clinical trial

**DOI:** 10.1097/MD.0000000000039585

**Published:** 2024-09-06

**Authors:** Yanlong Liu, Yihong Qian, Lilan Zhang, Shanliang Guo, Longcheng Fan, Mingsheng Zhang, Zhongyu Liu

**Affiliations:** a Department of Anesthesiology, Jiangxi Provincial People’s Hospital (The First Affiliated Hospital of Nanchang Medical College), Nanchang, P.R. China.

**Keywords:** ciprofol, etomidate, gastroscopy, propofol

## Abstract

**Background:**

To compare the efficacy and safety of ciprofol, propofol, propofol and etomidate mixture or ciprofol and etomidate mixture in patients undergoing painless gastroscopic anesthesia, and to explore the optimal plan to relieve the patient’s discomfort.

**Methods:**

A total of 120 patients scheduled for painless gastroscopy were randomly assigned to 4 groups: propofol (Group P), ciprofol (Group C), propofol-etomidate mixture (Group P-E), and ciprofol-etomidate mixture (Group C-E). The success rate of gastroscopy examination, patient satisfaction, incidence of injection pain, hemodynamic parameters, induction time, procedure time, the consumption of drugs, awakening time, and incidence of adverse events were evaluated.

**Results:**

All patients in the study successfully completed the gastroscopy. The satisfaction of patients in Group C-E was significantly higher than that in Group P (*P* < .05), but there was no statistical significance in the patient satisfaction among the other groups. Compared with Group P, the incidence of injection pain in Groups C and C-E significantly decreased (*P *< .05). There were no significant differences in the SBP, diastolic blood pressure, HR, and SpO_2_ among the 4 groups (*P* > .05). The awakening time of Group C was significantly longer than that of Groups P and P-E (*P* < .05), but there was no statistically significant difference in the awakening time of other groups.

**Conclusion:**

Ciprofol demonstrated efficacy in inducing sedation or anesthesia during painless gastroscopy that was similar to propofol, while exhibiting a comparable safety profile. Moreover, the combination of propofol and etomidate, as well as the combination of ciprofol and etomidate, were both shown to be equally safe and effective for this clinical application. These findings suggest that ciprofol can be considered as a safe and effective alternative for painless gastroscopy, and the ciprofol-etomidate mixture may be a better choice.

## 1. Introduction

Endoscopy is playing an increasingly important role in the diagnosis and treatment of digestive diseases with the evolution of endoscopic techniques. Gastroscopy is currently the most commonly used and reliable diagnosis and treatment method for the upper part of the gastrointestinal tract, and the number of patients undergoing gastroscopy is increasing year by year.^[1]^ However, people tend to feel tense, anxious, and fearful during this procedure, and painless treatment is the key to solving this problem. Painless treatment is a kind of human-oriented service, where patients can get physical and mental relaxation. It can alleviate or eliminate the pain and fear of patients in the process of treatment and improve patient satisfaction with treatment. Painless, safety, and comfort are inevitable trends in the development of anesthesia technology.^[2–4]^

Propofol is a commonly used sedative in clinical practice due to its rapid onset, rapid recovery, and minimal side effects. It provides excellent anesthesia for short surgeries and is highly favored by anesthesiologists.^[5]^ However, one significant drawback of propofol is the occurrence of painful injection during its administration for painless gastroscopy.^[6]^ Researchers have focused on optimizing propofol administration techniques to minimize injection pain, such as using a slow infusion rate, premedication with lidocaine, or combining it with other agents to enhance patient comfort. Hsu et al^[7]^ investigated the effects of different target concentrations of propofol in patients with painless endoscopy via a target-controlled infusion system, showing that the incidence of cardiopulmonary events (including hypotension, bradycardia, hypoxemia, etc.) was significantly positively correlated with the depth of anesthesia and the dosage of propofol.

Ciprofol is a novel intravenous anesthetic drug that has been introduced in recent years.^[8,9]^ It is considered an analog of propofol and shares a similar chemical structure. Ciprofol retains the advantages of propofol while improving its disadvantages.^[10]^ Clinical experiments have shown that ciprofol has a higher safety margin with a therapeutic index 2.4 times greater than propofol, indicating increased safety. It also exhibits 4 to 5 times the potency of propofol, highlighting its improved effectiveness.^[11,12]^ Studies have compared ciprofol to propofol in terms of deep sedation during gastroscopy and colonoscopy procedures, and ciprofol has been found to be non-inferior to propofol in terms of successful sedation with no significant adverse events reported.^[12]^

Etomidate is another sedative hypnotic drug commonly used in anesthesia induction for painless gastroscopy. It has stable anesthesia, a quick onset, and rapid recovery. Compared to propofol, etomidate has minimal effects on the respiratory and cardiovascular systems of patients.^[13,14]^ However, etomidate is rarely used alone due to potential complications such as muscle tremor, muscle stiffness, and postoperative nausea and vomiting.^[15,16]^

The co-administration of propofol and etomidate is a widely practiced approach in clinical settings. It has been shown to reduce hemodynamic fluctuations compared to other medications.^[17]^ A systematic review and meta-analysis of 15 studies have demonstrated that combining propofol and etomidate improves adverse reactions and provides significant benefits compared to using either agent alone, making it a safer and more effective option.^[18]^ The propofol-etomidate mixture offers enhanced patient comfort and improved sedation outcomes during gastroscopy and other procedures. However, there is relatively little research on the effectiveness of the ciprofol-etomidate mixture in gastroscopy.

To observe its further effect, a randomized, double-blind controlled clinical trial was conducted to compare the efficacy and safety of ciprofol, propofol, propofol and etomidate mixture or ciprofol and etomidate mixture in patients undergoing painless gastroscopic anesthesia, and to explore the optimal plan to relieve the patient’s discomfort. The findings of this study would provide valuable insights for selecting the most suitable anesthetic procedure for painless gastroscopy.

## 2. Methods

### 2.1. Ethics and trial registration

The Ethics Committee of Jiangxi Provincial People’s Hospital approved the protocol (Ethics Number: [2022]09), and the study was registered in the Chinese Clinical Trial Registry (ChiCTR2200066323). All participants signed the informed consent. The full trial protocol can be accessed at the following website: www.chictr.org.cn.

A total of 120 patients who underwent painless gastroscopy in Jiangxi Provincial People’s Hospital from December 2022 to February 2023 were selected in this study and randomized into 4 groups by the double-blind method.

### 2.2. Inclusion and exclusion criteria

Inclusion criteria: The current study included patients aged 18 to 60 years with an ASA (American Society of Anesthesiologists) physical status of I or II. All patients were required to provide signed informed consent.

Exclusion criteria were as follows: no escort or guardian; hearing and vision abnormalities; a history of mental illness, long-term use of psychotropic drugs and chronic sedative drugs; neuromuscular disorders; difficult airway; allergy to or contraindication for the drugs; and participation in clinical trials of other drugs within 30 days.

### 2.3. Randomization and blinding

Propofol (20 mL:200 mg, Yangtze River Pharmaceutical Group, Taizhou, China), ciprofol (20 mL:50 mg, Haisco Pharmaceutical Group, Xingcheng, China), and etomidate (10 mL:20 mg, Jiangsu Enhua Pharmaceutical Co., Ltd., Xuzhou, China) were all formulated 20 mL as white emulsions, making them visually indistinguishable. This meticulous formulation ensured successful blinding of both the researchers and the patients, preventing them from discerning the administered medications. A random sequence without stratification was generated by a computer and sealed with consecutively numbered envelopes to conceal the random assignment. Patients were randomly assigned to 4 groups using the envelope method to receive propofol (Group P), ciprofol (Group C), 0.2% etomidate 20 mg plus 1% propofol 100 mg at a volume ratio of 1:1 (Group P-E), 0.2% etomidate 20 mg plus 0.25% ciprofol 25 mg at a volume ratio of 1:1 (Group C-E), with 30 patients in each group. All the above operations were conducted by nurses, and the researchers and patients are blind.

### 2.4. Study interventions

After routine gastroscopy preparation, fasting for 6 hours, and no drinking for 4 hours before the procedure, the patients arrived at the endoscopy center and were asked about basic information. If the patients met the inclusion criteria, they signed an informed consent form. Emergency materials were prepared, including an anesthesia machine, endotracheal intubation kit, laryngoscope, aspirator, etc. All patients took the left recumbent position in the operating room, with standard monitoring that included noninvasive blood pressure, pulse oxygen saturation (SpO_2_), capnography and electrocardiogram. All patients inhaled oxygen using the nasal catheter (2 L/min). Then the peripheral venous channel was opened. Then, the patients will be randomly allocated into the propofol group (Group P), ciprofol group (Group C), etomidate propofol mixture group (Group P-E), or etomidate ciprofol mixture group (Group C-E) according to the information in the envelopes.

The drugs were prepared by a nurse anesthetist who was not involved in data collection. Each of the 4 groups received a 20 mL emulsion. In Group P, the emulsion contained 200 mg of propofol. In Group C, the emulsion contained 50 mg of ciprofol. Group P-E received a mixture of 10 mL containing 100 mg of propofol combined with 10 mL containing 20 mg of etomidate. Group C-E received a mixture of 10 mL containing 25 mg of ciprofol combined with 10 mL containing 20 mg of etomidate. Patients were given 0.2 mL/kg slow intravenous infusion (the infusion speed was about 10 s/2 mL). Gastroscopy can be performed in all 4 groups after the loss of consciousness, the disappearance of eyelash reflex, and modified observer’s assessment of alertness/sedation score (MOAA/S) ≤ 1. Gastroscopy was performed by skilled gastroenterologists with more than 10 years of experience in gastroscopy. If the patient had intraoperative laryngeal adductor reflex^[11]^ or body movement, the original corresponding induction drug 0.02 to 0.04 mL/kg could be added, which can be repeated. If the heart rate (HR) was lower than 50 beats/min, appropriate atropine (0.3–0.5 mg) should be injected intravenously. If systolic blood pressure (SBP) was lower than 80 mm Hg or 30% lower than before surgery, rapid intravenous supplementation of Ringer’s lactate solution was preferred, and norepinephrine can be given intravenously 4 to 8 µg/time if necessary, which can be repeated. In case of respiratory depression during gastroscopy, characterized by capnography indicating inadequate ventilation, hypoxemia (SpO_2_ < 90%, duration > 10 seconds), or apnea (no breathing > 15 seconds), the oxygen delivery concentration should be adjusted or jaw support provided. If these measures are ineffective, the endoscopist should withdraw the endoscope and provide assisted ventilation through jaw support or application of a pressure oxygen mask. In case of postoperative nausea and vomiting, patients with mild symptoms may not need to be treated, but patients with severe symptoms may be given symptomatic treatment of tropisetron. Injection pain was defined when the patient complained of pain or showed limb contraction during the intravenous injection.

### 2.5. Outcome

The main outcomes of this study included the success rate of gastroscopy examination, patient satisfaction, and injection pain. SBP, diastolic blood pressure, HR, and peripheral capillary oxygen saturation (SpO_2_) were recorded at different time points, including the time before anesthesia (T0), after the first administration (T1) and at the end of the examination (T2). Induction time, procedure time, drugs consumption, awakening time, dream experience, muscle tremor and body movement were also recorded.

### 2.6. Statistical analysis

PASS 11.0.7 (NCSS, LCC, Kaysville) was used for sample size calculation. Based on the results of clinical experiments,^[19]^ the incidence of injection pain during painless endoscopy treatment was found to be 6.3% for ciprofol (0.5 mg/kg) and 45.2% for propofol (2 mg/kg). With a one-sided type I error (α) of 0.025 and a power (1–β) of 0.9, it was determined that 23 subjects per group would be sufficient to detect differences in injection pain incidence. Accounting for a potential dropout rate of 20%, a total of 30 subjects per group were intended to be recruited.

SPSS 22.0 statistical software (IBM Corp., Armonk) was used to analyze the data collected in the study. Normal distribution measurement data were expressed as mean ± standard deviation (𝑥̅ ± s). Intra-group comparisons were conducted using repeated measures analysis of variance, while inter-group comparisons were performed using one-way analysis of variance. Categorical data were compared using the Fisher’s exact test or Chi-square test. The difference was statistically significant with *P* < .05.

## 3. Results

Between December 2022 and February 2023, 150 patients underwent eligibility assessment, with 30 patients excluded prior to random allocation. A total of 120 patients were randomly assigned, with 30 in each group, as detailed participant information is shown in guidelines flow diagram. All enrolled patients successfully completed the gastroscopy without any dropouts or incomplete procedures.

### 3.1. Demographic characteristics among the 4 groups

A total of 120 patients were assessed with an average age of 46.37 ± 11.19 years. Table 1 indicates their demographic characteristics. There were no significant differences in the baseline characteristics among the 4 groups (*P* > .05), Table 1

**Table 1:** Comparison of demographic data in patients (n = 30).

	Group P	Group C	Group P-E	Group C-E	*P*
Age, years, 𝑥̅± s	45.00 ± 12.96	45.63 ± 10.07	50.07 ± 8.70	44.77 ± 12.23	.216
Male, n (%)	18 (60.0)	13 (43.3)	12 (40.0)	14 (46.7)	.428
BMI, kg/m^2^, 𝑥̅± s	23.69 ± 2.62	23.00 ± 2.86	22.36 ± 2.32	22.94 ± 2.50	.265

BMI = body mass index.

### 3.2. Success rates of gastroscopy among the 4 groups

The success rate of gastroscopy was 100% among the 4 groups. The sedation success rate of insertion for gastroscopy was 100% among the 4 groups.

### 3.3. Anesthesia-related indices among the 4 groups

The patient satisfaction of Group C-E was significantly higher than that of Group P (*P* < .05), but there was no statistical significance in the patient satisfaction among the other groups.

Compared with Group P, the incidence of injection pain in Groups C and C-E significantly decreased (*P* < .05), the pain intensity was lower.

There was no statistical significance difference in the induction dosage (mL), additional dosage (mL), and total dosage (mL) among the 4 groups.

The awakening time of Group C was significantly longer than that of Groups P and P-E (*P* < .05), but there was no significant difference in the awakening time of other groups.

There was no statistical significance in the induction time and procedure time among the 4 groups (Table 2).

**Table 2:** Comparison of anesthesia-related indices in patients (n = 30).

	Group P	Group C	Group P-E	Group C-E	*P*
Induction dosage, mL, 𝑥̅ ± s	13.76 ± 2.05	13.30 ± 2.11	12.47 ± 2.16	13.28 ± 2.62	.166
Induction time, s, 𝑥̅ ± s	63.77 ± 12.86	60.73 ± 16.12	55.67 ± 15.24	56.33 ± 12.77	.099
Additional dosage, mL, 𝑥̅ ± s	6.87 ± 4.96	4.53 ± 3.40	5.52 ± 4.94	6.32 ± 4.33	.202
Total dosage, mL, 𝑥 ®± s	20.63 ± 5.22	17.83 ± 3.47	17.99 ± 5.14	19.69 ± 4.50	.062
Procedure time, min, 𝑥̅ ± s	11.77 ± 7.31	11.47 ± 7.15	9.93 ± 6.37	12.07 ± 6.94	.643
Awakening time, min, 𝑥̅ ± s	6.57 ± 1.83	8.53 ± 2.78*	6.40 ± 2.36	8.30 ± 3.28	.002
Patient satisfaction (very satisfactory), n (%)	19 (63.3)	25 (83.3)	25 (83.3)	29 (96.7)†	.010
Injection pain, n (%)	14 (46.7)	5 (16.7)‡	7 (23.3)	2 (6.7)‡	.002
Mild	5	4	3	1	
Moderate	8	1	4	1	
Severe	1	0	0	0	

Compared with Group P,

* *P* < .05,

† *P* < .05,

‡ *P* < .05.

### 3.4. Hemodynamic indicators and SpO_2_ among the 4 groups

There were no significant differences in the SBP, diastolic blood pressure, HR, and SpO_2_ among the 4 groups (*P* > .05), Table 3.

**Table 3:** Comparison of hemodynamic indicators in patients (n = 30).

Time		Group P	Group C	Group P-E	Group C-E	*P*
Before anesthesia (T0)	HR, beats/min, 𝑥̅ ± s	82.40 ± 13.06	83.47 ± 13.50	79.17 ± 12.60	77.03 ± 13.00	.208
	SBP, mm Hg, 𝑥 ®± s	125.47 ± 21.37	122.77 ± 18.38	128.73 ± 17.77	121.97 ± 20.33	.532
	DBP, mm Hg, 𝑥 ®± s	68.53 ± 11.49	68.20 ± 15.54	71.47 ± 12.25	70.13 ± 12.70	.753
	SpO_2_, %, 𝑥̅ ± s	99.47 ± 1.07	99.70 ± 0.75	99.73 ± 0.58	99.50 ± 0.68	.453
After the first administration (T1)	HR, beats/min, 𝑥̅ ± s	83.60 ± 10.29	81.63 ± 12.84	77.03 ± 10.73	77.00 ± 11.56	.058
	SBP, mm Hg, 𝑥 ®± s	105.47 ± 18.25	106.50 ± 17.94	107.90 ± 18.06	104.83 ± 15.79	.913
	DBP, mm Hg, 𝑥̅ ± s	60.70 ± 11.96	62.10 ± 17.59	64.53 ± 12.06	62.13 ± 12.96	.756
	SpO_2_, %, 𝑥̅ ± s	99.40 ± 0.97	99.90 ± 0.40	99.67 ± 0.88	99.87 ± 0.43	.058
At the end of the examination (T2)	HR, beats/min, 𝑥̅ ± s	74.27 ± 8.12	74.00 ± 9.40	73.50 ± 12.14	72.20 ± 11.81	.876
	SBP, mm Hg, 𝑥̅ ± s	99.43 ± 11.02	106.53 ± 18.66	108.00 ± 16.90	107.07 ± 14.04	.126
	DBP, mm Hg, 𝑥 ®± s	58.53 ± 8.24	62.37 ± 12.21	63.70 ± 10.53	61.57 ± 10.77	.279
	SpO_2_, %, 𝑥̅ ± s	99.63 ± 0.67	99.93 ± 0.25	99.83 ± 0.38	99.90 ± 0.44	.112

DBP = diastolic blood pressure, HR = heart rate, SBP = systolic blood pressure, SpO_2_ = oxygen saturation.

### 3.5. Incidence of dream, muscle tremor and body movement among the 4 groups

In this study, there was no statistical significance in the dream experience and the occurrence of muscle tremor after anesthesia induction among the 4 groups. The incidence of body movement in Group C-E was significantly lower than that of Group P (*P* < .05) (Table 4).

**Table 4:** Comparison of incidence of dream, muscle tremor and body movement in patients (n = 30).

	Group P	Group C	Group P-E	Group C-E	*P*
Dream, n (%)	8 (26.7)	8 (26.7)	7 (23.3)	6 (20.0)	.965
Muscle remor, n (%)	2 (6.7)	4 (13.3	4 (13.3)	5 (16.7)	.766
Body movement, n (%)	13 (43.3)	6 (20.0)	8 (26.7)	3 (10.0)*	.025

Compared with Group P,

* *P* < .05.

## 4. Discussion

Gastroscopy is a procedure for the diagnosis of diseases of the upper digestive tract. Although it is a noninvasive operation, its invasive procedure can cause psychological stress reaction strongly on patients and low clinical compliance, thus leading to surgery failure and increasing medical costs. The painless gastroscopy technique can significantly improve the satisfaction of anesthesiologists and the comfort of patients. At present, light/moderate sedation has become the clinical consensus of gastroenterology anesthesia at home and abroad.^[20]^

This study demonstrated that there was no significant difference in gastroscopy success rate between the 4 groups: 2 mg/kg propofol, 0.5 mg/kg ciprofol, 1 mg/kg propofol plus 0.2 mg/kg etomidate, and 0.25 mg/kg ciprofol plus 0.2 mg/kg etomidate. All 4 groups successfully completed the gastroscopy procedure, consistent with previous research findings.^[21,22]^ Furthermore, there were no statistically significant differences in the induction dose (mL), additional dose (mL), and total dose (mL) of the 4 groups during the short-term, painless gastroscopy procedures. However, when the dosages were converted to milligrams (mg), the actual amounts of drugs used in Group P and Group P-E were notably higher compared to Group C and Group C-E. This suggests that the dosage of ciprofol was equivalent to approximately 1/5 to 1/4 of the propofol dosage required to achieve the same anesthetic effect. Although the recovery time was prolonged with the use of ciprofol, its safety profile appeared to be improved compared to propofol. Ciprofol,^[12]^ with similar mechanisms of action to propofol, is a γ-aminobutyric acid (GABA) receptor agonist with a short-acting sedative property. It enhanced GABA receptor-mediated ion channels, resulting in chloridion-internal flow. However, studies have shown that the binding ability of ciprofol to the GABA receptor is higher than that of propofol. Except for GABA, the inhibition rate of ciprofol on other targets was less than 51% (10 μM), and only 1/5 concentration of ciprofol could achieve the same effect of the GABA receptor-mediated cell currents as propofol.

The higher patient satisfaction observed in Groups P-E, C-E, and C compared to Group P is likely attributed to reduced injection pain. Our findings revealed higher incidence of injection pain in Group P (14/46.7%) compared to Groups C (5/16.7%), P-E (7/23.3%), and C-E (2/6.7%). While Group P experienced 14 cases of pain, including 1 severe and 8 moderate cases, the remaining 3 groups primarily reported mild to moderate pain. These results indicate that propofol alone administration resulted in a significantly higher incidence and severity of injection pain compared to Group C, P-E, and C-E. It was generally believed that propofol injection pain was related to the concentration of water phase in the fat emulsion.^[3]^ Ciprofol, dissolved in medium/long-chain triglyceride fat emulsion, has a higher potency and causes less injection pain due to its molecular structure and lipid solubility, despite having the same water phase concentration as propofol in a 1% fat emulsion. The reduction in injection pain associated with propofol-etomidate mixtures is attributed to the vasodilatory effect of etomidate, which partially counteracts the irritant properties of propofol. While this combination generally mitigates injection pain, a small proportion of patients may still experience discomfort. Wang^[23]^ investigated the anesthetic effect of etomidate and propofol mixture in painless gastrointestinal endoscopy, finding that the incidence of injection pain in the E-P group (etomidate-propofol group) was lower than that in the P group (propofol group), and the induction time, procedure time and awakening time of the 2 groups were similar, which was consistent with the result of this study.

The awakening time of Group C was significantly longer than that of Groups P and P-E (*P* < .05), while no significant difference was observed in the awakening time between the other groups. This finding aligns with the results of Chen et al,^[24]^ suggesting that the longer half-life of ciprofol may contribute to the prolonged awakening time. The longer half-life of ciprofol compared to propofol is likely attributed to differences in its pharmacokinetic properties, such as altered distribution patterns, slower metabolism, or greater protein binding, which collectively contribute to the prolonged elimination of the drug from the body. These pharmacokinetic factors result in a slower decline in ciprofol concentrations in the brain, leading to the observed delays in patient awakening times.

This study found no statistically significant differences in systolic blood pressure, diastolic blood pressure, heart rate, or oxygen saturation (SpO_2_) at time points T0, T1, and T2 among the 4 groups. The systolic and diastolic blood pressures in all 4 groups were significantly decreased at T1 and T2 compared to T0, which is consistent with the findings of previous studies.^[19,24]^ This is likely attributed to the reduced vascular tone and peripheral vasodilation that occur following drug administration, leading to the observed decline in blood pressure. The heart rates at T1 did not differ greatly from T0 for all groups, while the heart rates at T2 were notably lower compared to T0 and T1. This relatively stable heart rate response during the early phase after drug administration may be associated with patient anxiety, blood pressure depression and injection pain, which could mask the expected heart rate changes.

In addition, short-acting anesthetics may be associated with a higher incidence of dream experiences.^[25]^ This study found that although there was no significant difference in the dream experience and the occurrence of muscle tremor after anesthesia induction among the 4 groups, more than 26% of patients with ciprofol had dreams. Although the dreamer was not able to remember much of the content of the dream, most of the dreams were pleasant, simple, and emotionally positive, and very few dreams were emotionally negative, which may be associated with the enhancement of emotional memory consolidation through GABA receptor-mediated ion channels.^[26]^ The incidence of muscle tremors was the highest in Group C-E, which was more than 16%. Etomidate is a drug with little effect on the circulatory system and many adverse events, which is rarely used alone. Myoclonus is one of its main adverse events. Jae^[27]^ proposed that etomidate induced myoclonus in a dose-dependent manner, and high doses of etomidate could inhibit subcortical activity. Inhibition of the spinal cord or the cerebral cortex may result in the derepression of subcutaneous structures rather than the epileptic focus. Recent studies have shown that the anesthetic effect of etomidate was associated with enhanced tyrosine inhibition of GABA uptake and further inhibition of Ca^2+^ and Na^+^ channels.^[28]^ Teng found in their study that a low dose of ciprofol (0.2 mg/kg) was associated with a higher incidence of muscle fasciculation.^[19]^ These symptoms may be related to the administration of low doses of anesthetic drugs, similar to our study. Whether ciprofol has a synergistic effect with etomidate to induce muscle tremor needs to be further studied.

Despite the valuable information provided by this study regarding the selection of sedatives for painless gastroscopy, several limitations need to be considered. Firstly, the sample size was relatively small, with approximately 30 patients per group, which may affect the statistical power and generalizability of the results. Future studies could benefit from larger sample sizes to enhance the robustness of the findings. Secondly, the study focused only on short-term outcomes during the gastroscopy procedure, such as injection pain and patient satisfaction, without evaluating long-term indicators such as postoperative complications and recovery. It is recommended to incorporate longer follow-up periods in future research to comprehensively assess the safety and efficacy of these drugs. Thirdly, the study was conducted in a single center, which may introduce geographic limitations. Future research involving multiple centers would improve the external validity of the results. In conclusion, while this study provides valuable insights into the selection of sedatives for painless gastroscopy, these limitations should be further explored and addressed in subsequent studies to validate the clinical utility of these novel drugs.

## 5. Conclusion

In conclusion, based on the findings of this study, Ciprofol demonstrated a safety profile comparable to propofol and exhibited similar efficacy in inducing sedation or anesthesia during painless gastroscopy. Ciprofol exhibited significant advantages in terms of injection pain reduction. The combination of etomidate with ciprofol showed improved patient satisfaction, reduce body movement and overall patient comfort. These findings suggest that ciprofol can be considered as a safe and effective alternative for painless gastroscopy, and the ciprofol-etomidate mixture may be a better choice. Further research and clinical trials are warranted to validate these findings and explore the full potential of Ciprofol in gastroscopic procedures.

## Author contributions

**Conceptualization:** Mingsheng Zhang.

**Data curation:** Yanlong Liu, Yihong Qian, Lilan Zhang.

**Formal analysis:** Lilan Zhang.

**Funding acquisition:** Mingsheng Zhang.

**Investigation:** Yanlong Liu, Lilan Zhang.

**Methodology:** Yanlong Liu, Lilan Zhang.

**Project administration:** Yanlong Liu, Shanliang Guo, Longcheng Fan, Mingsheng Zhang.

**Resources:** Yanlong Liu, Shanliang Guo, Longcheng Fan.

**Software:** Shanliang Guo.

**Supervision:** Yihong Qian, Mingsheng Zhang.

**Validation:** Mingsheng Zhang.

**Visualization:** Lilan Zhang.

**Writing – original draft:** Yanlong Liu.

**Writing – review & editing:** Zhongyu Liu.
